# Cardiac implantable electronic device surgery with interruption of warfarin forgoing post-operative bridging therapy in patients with moderate or high thromboembolic risks

**DOI:** 10.1186/s12959-021-00279-6

**Published:** 2021-04-29

**Authors:** Andrew Kei-Yan Ng, Pauline Yeung Ng, Eva Wai-Ying Tam, Chung-Wah Siu, Katherine Fan

**Affiliations:** 1 Cardiac Medical Unit, Grantham Hospital, 125 Wong Chuk Hang Road, Hong Kong SAR, China; 2 Department of Adult Intensive Care, Queen Mary Hospital, 102 Pokfulam Road, Hong Kong SAR, China; 3 Division of Respiratory and Critical Care Medicine, Department of Medicine, Li Ka Shing Faculty of Medicine, The University of Hong Kong, Hong Kong SAR, China; 4 Department of Medicine, Queen Mary Hospital, The University of Hong Kong, Hong Kong SAR, China

## Abstract

**Background:**

For patients taking warfarin and undergoing pacemaker or implantable cardioverter-defibrillator surgery, clinical evidence and guidelines support continuation of warfarin therapy, as opposed to interruption of warfarin therapy with heparin bridging. Interruption of warfarin without post-operative bridging therapy may be a feasible alternative but data is sparse.

**Methods:**

This is a single-arm observational study including adults who had interruption of warfarin therapy without post-operative bridging therapy for cardiac implantable electronic device (CIED) surgery performed between 2010 and 2019 in a tertiary referral hospital. The primary outcome was a composite of all-cause mortality, arterial or venous thromboembolic events. The secondary outcomes were clinically significant device-pocket hematoma and other procedural complications.

**Results:**

Of the 411 patients analysed including 257 patients (62.5%) who had mechanical heart valves, the primary outcome developed in 5 (1.2%) patients within 30 days after surgery, including death in 3 (0.7%) patients, transient ischemic attack in 1 (0.2%) patient and non-CNS embolism in 1 (0.2%) patient. Clinically significant hematomas occurred in 24 (5.8%) patients, including 15 (3.7%) requiring additional interruption of anti-coagulation and 6 (1.5%) requiring clot evacuation. Other procedural complications and bleeding events were rare (< 1%).

**Conclusions:**

Warfarin interruption without post-operative bridging therapy for CIED surgery was associated with low thromboembolic risks and acceptable bleeding risk. Randomized controlled trials are required to formulate an optimal approach to anti-coagulation management.

## Background

Annually, an estimated 1.25 million pacemaker and 410,000 implantable cardioverter defibrillator (ICD) operations are performed [1]. Between 14 and 37% of patients undergoing cardiac implantable electronic device (CIED) surgeries are on long-term anti-coagulation therapy, and the peri-procedural management of anti-coagulation presents a dilemma to physicians [2–5]. Interruption of anti-coagulation therapy can transiently increase the risks of thromboembolic events, but continuing anti-coagulation therapy can increase the risk of surgical site hematoma formation. Importantly, there is an association between hematoma formation and subsequent device system infection. For example, patients with device infections were 20-fold and 8-fold more likely to have had postoperative hematomas in the REPLACE registry and the BRUISE CONTROL INFECTION study, respectively [6, 7]. CIED infections frequently necessitate complete system removal, and are associated to increased morbidity, mortality and cost [8].

Randomized trials, most notably the BRUISE CONTROL (INFECTION) study, showed that continuation of warfarin was superior to the previous practice of interruption of warfarin therapy with heparin bridging [9–11]. However, a strategy of interruption of warfarin without heparin bridging have not been examined in these trials, and there is growing evidence that this may be a better peri-operative strategy. For example, in the BRIDGE randomized trial, patients with atrial fibrillation (AF) who had warfarin therapy interrupted for elective surgeries (not including CIED surgeries) without heparin bridging benefited from fewer bleeding complications with no excessive thromboembolic risks [12].

We sought to examine the risks of thromboembolic events and clinically significant device pocket hematoma with a strategy of warfarin interruption without post-operative heparin bridging in moderate or high risk patients undergoing CIED surgeries.

## Methods

### Study population and design

Data from consecutive patients who were taking chronic warfarin therapy and underwent CIED surgery between January 1, 2010 and December 31, 2019 at Grantham Hospital, Hong Kong were reviewed. The study was approved by the Institutional Review Board of the University of Hong Kong / Hospital Authority, and a waiver of informed consent was granted.

We included all adult patients (18 years of age or older) who were on chronic warfarin therapy, underwent CIED surgeries (including pacemaker implantations, cardiac resynchronization therapy, ICD implantations, and generator replacements) and had interruption of warfarin therapy without any post-operative bridging therapy with heparin or any other anti-coagulant. Pre-operative heparin bridging was allowed until 12 h before surgery. We excluded patients who underwent lead extractions or leadless pacemaker implantation within the same index procedure, had a warfarin interruption period of less than 24 h, or lost to follow-up within 6 months after the index procedure.

### Definitions of exposure and outcome variables

The primary outcome was all-cause mortality, and a composite of perioperative thromboembolic events including transient ischemic attack (TIA), ischemic stroke, peripheral artery and venous thromboembolism, within 30 days after CIED surgery.

The secondary outcome was a composite of clinically significant device-pocket hematoma: defined as any surgical site hematoma requiring repeated surgery and/or blood transfusion, or resulting in prolongation of hospitalization, or requiring additional interruption of oral anticoagulation therapy. Prolongation of hospitalization was defined as extended hospitalization for at least 24 h after the index surgical procedure or any re-hospitalization, primarily for management of hematoma. Additional interruption of anticoagulation therapy was defined as reversal or intentional delayed resumption of warfarin therapy for at least 24 h, primarily due to surgical site hematoma. All events were confirmed by chart review by two independent investigators.

The total warfarin interruption period was defined as the duration from 12 h after the last dose of unfractionated or low molecular weight heparin, until the first international normalized ratio (INR) of > 1.8, or when the INR is no longer tested on a daily basis, as it reflects attainment of target INR values.

### Routine procedural protocol

As per our routine protocol for CIED surgeries, warfarin was discontinued 3–4 days prior to and resumed 0–1 day after the procedure. Pre-operative low molecular weight heparin (LMWH) was given to patients with high thrombotic risks (e.g., those with mechanical heart valves, mitral stenosis of at least moderate severity, or CHAD_2_S_2_-VASc score ≥ 2) when the INR fell below 1.8–2.0, and continued until 12 h before the procedure. Post-operative LMWH was not given. Aspirin and P2Y12 inhibitors were continued if indicated. All patients received intravenous prophylactic antibiotics. A pressure dressing was routinely applied to the surgical wound postoperatively and left in place overnight.

### Statistical analysis

Data are presented as mean with standard deviation or median with interquartile range as appropriate. Descriptive analysis of baseline characteristics, procedural details, primary and secondary outcomes were reported for the entire cohort and stratified by presence of any mechanical heart valves. In the exploratory analysis, we used a self-controlled case-series design to explore the association between CIED surgery and thromboembolic events, by defining the “risk interval” as the first 30 days after CIED surgery and the “control interval” as 12 months before and 11 months after the risk interval [13]. Data management and statistical analyses were performed in Stata software (StataCorp/MP version 16).

## Results

### Patients and characteristics

**Fig. 1:**
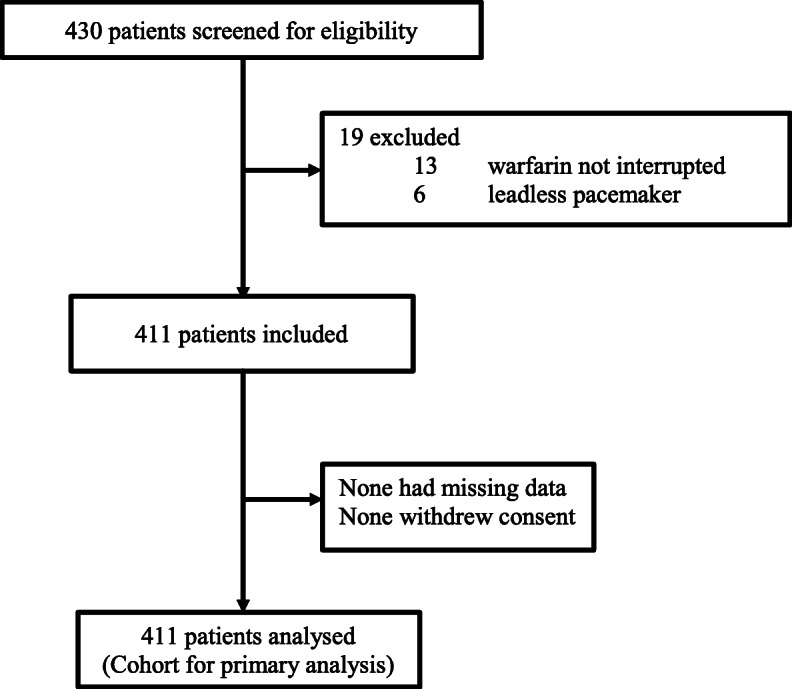
Study profile

Between January 2010 to December 2019, a total of 430 patients were considered for inclusion: 19 (4.4%) were excluded due to any of the exclusion criteria (Fig. 1). No patients were lost to follow-up. The baseline characteristics of the patients are shown in Tables 1 & 2. Of the 411 patients included in analysis, 257 (62.5%) had at least one mechanical prosthetic valve. Among the patients without mechanical prosthetic valve, 132 (85.7%) were taking warfarin because of atrial fibrillation or atrial flutter. Procedural details and peri-operative anti-coagulation management are shown in Table 3. The mean INR on the day of surgery was 1.44 ± 0.22 and the mean duration without any therapeutic anti-coagulation was 4.04 ± 2.04 days. Pre-operative heparin bridging was given in 57.2% of patients.

**Table 1:** Baseline characteristics of patients, stratified by presence of mechanical valves

	All patients		Mechanical valve		No mechanical valve	
	*N* = 411		*N* = 257		*N* = 154	
Age - yr ± S.D.	66.67	± 11.92	65.56	± 10.04	65.85	± 14.56
Age > 65 - no. (%)	208	50.6%	129	50.2%	79	51.3%
Male sex - no. (%)	178	43.3%	90	35.0%	88	57.1%
Medical history - no. (%)						
Rheumatic heart disease	251	61.1%	224	87.2%	27	17.5%
Mitral stenosis (native valve)	13	3.2%	0	0.0%	13	8.4%
Tricuspid regurgitation (at least moderate)	156/277	56.3%	111/186	59.7%	45/91	49.5%
Prior transient ischemic attack	21	5.1%	16	6.2%	5	3.3%
Prior ischemic stroke	54	13.1%	32	12.5%	22	14.3%
Prior non-CNS embolic event	3	0.7%	1	0.4%	2	1.3%
Hypertension	159	38.7%	89	34.6%	70	45.5%
Diabetes mellitus	100	24.3%	58	22.6%	42	27.3%
Chronic kidney disease (eGFR< 30 ml/m^2^)	68	16.5%	35	13.6%	33	21.4%
Cardiomyopathy	112	27.3%	37	14.4%	75	48.7%
Coronary artery disease	61	14.8%	19	7.4%	42	27.3%
Percutaneous coronary intervention	30	7.3%	6	2.2%	24	15.6%
Coronary-artery bypass surgery	15	3.7%	7	2.7%	8	5.2%
Left ventricular ejection fraction < 50%	147	35.8%	74	28.8%	73	47.4%
Left ventricular ejection fraction - percentage point ± S.D	50.75	± 17.66	53.74	± 15.92	45.75	± 19.27

Abbreviation: *S.D.* standard deviation

**Table 2:** Baseline characteristics of patients, stratified by presence of mechanical valves

	All patients		Mechanical valve		No mechanical valve	
	*N =* 411		*N =* 257		*N =* 154	
Indication for warfarin therapy - no. (%)						
Mechanical heart valve replacement (any)	257	62.5%	257	100.0%		
Mechanical mitral valve replacement	212	51.3%	212	82.5%		
Mechanical aortic valve replacement	121	29.0%	121	47.1%		
Mitral stenosis (native valve)	13	3.2%			13	8.4%
Atrial fibrillation or atrial flutter	318	77.4%	186	72.4%	132	85.7%
CHAD2S2-VASc (for non-valvular AF; *N =* 119) - mean ± S.D.					3.34	± 1.93
CHAD2S2-VASc ≥2 (for non-valvular AF; *N =* 119)					96	80.7%
Deep vein thrombosis or pulmonary embolism	4	1.0%	0	0.0%	4	2.6%
Intracardiac thrombus	14	3.4%	0	0.0%	14	9.1%
Medications - no. (%)						
Any anti-platelet therapy	69	16.8%	20	7.8%	49	31.8%
Aspirin	68	16.6%	20	7.8%	48	31.2%
P2Y12 inhibitor	5	1.2%	1	0.4%	4	2.6%
Statin	153	37.2%	78	30.4%	75	48.7%
ACE inhibitor or ARB	215	52.3%	117	45.5%	98	63.6%
Beta-blocker	220	53.5%	118	45.9%	102	66.2%
Diuretics	312	75.9%	196	76.3%	116	75.3%
Pre-operative low molecular weight heparin	235	57.2%	181	70.4%	54	35.0%

Abbreviations: *S.D.* standard deviation, *ACE* angiotensin converting enzyme, *ARB* angiotensin receptor blocker

**Table 3:** Baseline and intraoperative characteristics of patients

	All patients		Mechanical valve		No mechanical valve	
	*N =* 411		*N =* 257		*N =* 154	
CIED procedure - no. (%)						
New implant						
Pacemaker						
Single or dual chamber	131	31.9%	81	31.5%	50	32.5%
Cardiac resynchronization therapy	14	3.4%	9	3.5%	5	3.3%
Implantable cardioverter-defibrillator						
Single or dual chamber	21	5.1%	6	2.3%	15	9.7%
Cardiac resynchronization therapy	27	6.6%	9	3.5%	18	11.7%
Device replacement or revision						
Pulse-generator change only	191	46.5%	129	50.2%	6	40.3%
Pulse-generator change with additional procedure	27	6.6%	23	9.0%	4	2.6%
INR on day of surgery - mean ± S.D.	1.44	± 0.22	1.44	± 0.21	1.43	± 0.24
Warfarin resumption after surgery -days						
Mean ± S.D.	0.46	± 1.01	0.23	± 0.51	0.84	± 1.45
Median, IQR	0	0–1	0	0–0	0	0–1
Anti-coagulation interruption period - days						
Mean ± S.D.	4.04	± 2.04	3.91	± 1.71	4.27	± 2.47
Median (IQR)	3	3–5	3	3–5	4	3–5

Abbreviations: *CIED* cardiac implantable electronic device, *S.D.* standard deviation, *IQR* interquartile range

### Primary and secondary outcomes

Of the 411 patients analysed, the primary composite outcome occurred in 5 (1.2%) patients within 30 days after surgery, including 3 (0.7%) deaths – one patient died of subarachnoid hemorrhage and two patients died of sudden cardiac arrest, 1 (0.2%) transient ischemic attack and 1 (0.2%) non-central nervous system (CNS) embolism. Clinically significant hematomas occurred in 24 (5.8%) patients, including 15 (3.7%) who required additional interruption of anti-coagulation and 6 (1.5%) who required clot evacuation. Other thromboembolic and bleeding events were rare. The study outcomes are detailed in Tables 4 and 5.

**Table 4:** Primary outcomes at 30 days after surgery

	All patients		Mechanical valve		No mechanical valve	
	*N =* 411		*N =* 257		*N =* 154	
Primary Outcome - no. (%)						
Death or any thromboembolic events	5	1.2%	4	1.6%	1	0.6%
Components of primary outcome						
Death from any cause	3	0.7%	2	0.8%	1	0.6%
Transient ischemic attack	1	0.2%	1	0.4%	0	0.0%
Ischemic stroke	0	0.0%	0	0.0%	0	0.0%
Non-CNS embolism	1	0.2%	1	0.4%	0	0.0%
Deep vein thrombosis	0	0.0%	0	0.0%	0	0.0%
Pulmonary embolism	0	0.0%	0	0.0%	0	0.0%
Valve thrombosis	0	0.0%	0	0.0%	0	0.0%

Abbreviation: *CNS* central nervous system

**Table 5:** Secondary outcomes at 30 days after surgery

	All patients		Mechanical valve		No mechanical valve	
	*N =* 411		*N =* 257		*N =* 154	
Secondary outcomes - no. (%)						
Clinically significant hematoma	24	5.8%	18	7.0%	6	3.9%
Hematoma prolonging hospitalization	24	5.8%	18	7.0%	6	3.9%
Hematoma requiring additional interruption of anti-coagulation	15	3.7%	9	3.5%	6	3.9%
Hematoma requiring evacuation	6	1.5%	4	1.6%	2	1.3%
Pneumothorax	0	0.0%	0	0.0%	0	0.0%
Hemothorax	0	0.0%	0	0.0%	0	0.0%
Cardiac tamponade	0	0.0%	0	0.0%	0	0.0%
Lead dislodgement	4	1.0%	3	1.2%	1	0.7%
Infection related to device system	4	1.0%	3	1.2%	1	0.7%
Myocardial infarction	0	0.0%	0	0.0%	0	0.0%
Major bleeding unrelated to CIED	2	0.5%	2	0.8%	0	0.0%

Abbreviation: *CIED* cardiac implantable electronic device

The primary endpoint occurred at similar frequencies in the mechanical valve compared with the no mechanical valve group (unadjusted risk ratio 2.40 [95% confidence interval, 0.27–21.20]; *P* = 0.66).

### Exploratory analysis

We identified 22 (5.4%) patients who had arterial or venous thromboembolic events during the control interval of 12 months before and between 1 to 12 months after CIED surgery. The risks of thromboembolic events were not significantly different during the risk interval and the control interval (risk ratio 3.08 [95% confidence interval 0.92–10.2]; *P* = 0.054).

## Discussion

In this cohort of patients on chronic warfarin therapy undergoing CIED surgery with moderate to high thromboembolic risks, a strategy of warfarin interruption without post-operative bridging therapy were found to have a relatively low risk of all-cause death or thromboembolism (1.2%) and an acceptable risk of device-pocket hematoma (5.8%). Importantly, we included more than 60% patients with mechanical prosthetic valves and showed that the rates of thromboembolism was comparable with patients without mechanical valves.

There is increasing evidence supporting continued warfarin therapy for CIED surgeries. In the BRUISE CONTROL trial including 668 patients with AF, continued warfarin therapy was superior with respect to risks of major bleeding and non-inferior to heparin bridging with respect to arterial thrombo-embolism [9]. This has led to a shift in European and American guidelines towards favoring continued warfarin therapy over interruption with bridging therapy for CIED surgeries [14–16]. However, there is limited published data on the efficacy and safety of the strategy of warfarin interruption and no post-operative bridging therapy used in our cohort.

According to a European survey, 9.4% patients undergoing CIED surgeries had warfarin interrupted for more than 24 h without bridging therapy, amid a lack of general consensus or clinical evidence [17]. Indirect clinical evidence supporting this practice stems from the setting of elective non-cardiac procedures, where randomized trials (including the BRIDGE trial) and cohort studies showed that warfarin interruption without bridging therapy was superior to with bridging therapy, with lower bleeding risks and no excess risks of thromboembolism [12, 18].. Moreover, in patients undergoing CIED implantations, post-operative LMWH and higher INR on day of CIED implantations were independent predictors of device-related hematoma in a case-control study [19]. Therefore, a strategy of warfarin interruption without post-operative bridging therapy has theoretical advantage in simultaneously addressing both risk factors. This naturally raises concern about thromboembolic risks during the period of warfarin interruption. The rationale of heparin bridging was to leverage the short half-life of LMWH (3–5 h) to maximize protection against thromboembolism during the pre-operative period, given the time period for INR normalization after warfarin interruption is widely variable [20, 21].

To our knowledge, our study is the largest cohort reporting the safety and efficacy in patients who had warfarin interruption for CIED surgeries without post-operative heparin bridging. Ahmed et al. reported data including 114 patients who had warfarin interruption for CIED implantations, and found significantly higher risks of TIA [22]. However pre-operative bridging therapy was not given and none of the patients had irreversible thromboembolic events. Another nation-wide registry included 150 patients with AF who had warfarin interruption for CIED implantations without bridging therapy, and both bleeding and thromboembolic events were very low (< 1%) [23]. A prematurely terminated trial randomized 171 patients and showed that events were similarly infrequent for reduced-dose warfarin vs warfarin interruption with LMWH bridging, including zero thromboembolic events [24]. Although observational in nature, our data suggests that the strategy under study conferred relatively low thromboembolic risks, which were not significantly higher than the study population’s baseline risks. These results should be considered hypothesis-generating for future randomized studies.

The risks of all-cause mortality and non-fatal thromboembolic event in our patients were 0.7 and 0.4% respectively, which were comparable with both arms in the BRUISE CONTROL trial (0 and 0% respectively for the heparin bridging arm, and 1.2 and 0.6% respectively for the continued warfarin arm) [9]. The risks of clinically significant hematoma in our patients were 5.8%, comparable with the reported 4.6% in a meta-analysis of 5978 patients receiving CIED with various combinations of anti-coagulant and/or anti-platelet therapy [25]. Specifically, the risks of bleeding complication in our cohort were much lower than the heparin bridging arm (16%) but numerically higher than the continued warfarin arm (3.5%) in the BRUISE CONTROL trial [9]. This could be attributed to the high portion of patients with valvular heart disease including more than half with at least moderate tricuspid regurgitation. Tricuspid regurgitation is associated with increased venous pressures and could adversely affect wound hemostasis. In addition, the main subcomponent of bleeding events in our patients was prolonged hospitalization secondary to management of hematoma. This endpoint could be affected by local discharge policies, as the pressure to discharge early from hospital is small in a non-insurance funded system. The other two subcomponents of bleeding events, hematoma requiring interruption of anti-coagulation (3.7%) and hematoma requiring evacuation (1.5%), were similar to the continued warfarin arm (3.2 and 0.6% respectively) and much lower than the heparin bridging arm (14.2 and 2.7% respectively) in the BRUISE CONTROL trial [9]. Nonetheless, comparisons across different patient cohorts are inherently limited, and a randomized trial is needed to compare the two strategies.

Importantly, our patients represented a cohort with moderate to high thromboembolic risks. We included 257 (62.5%) patients with mechanical prosthetic valves and a strategy of warfarin interruption without heparin bridging has never been reported in this population. The primary endpoint occurred at similar frequencies for the mechanical valve and no mechanical valve groups, providing preliminary findings that this anti-coagulation strategy in patients with mechanical valves may be considered.

The strengths of the present study include complete follow-up of all patients, large cohort size compared with previous studies that evaluated no bridging therapy, and inclusion of a sizeable proportion of patients with mechanical prosthetic valves whose outcomes with the current strategy have never been reported. The main limitation is the lack of a comparison group of either continued warfarin therapy or interruption of warfarin with bridging therapy. However, the findings arising from this study should serve to inform future randomized non-inferiority studies comparing our strategy of warfarin interruption without post-operative bridging therapy versus continued warfarin therapy.

## Conclusion

Warfarin interruption without post-operative bridging therapy for CIED surgery was associated with relatively low thromboembolic risks and bleeding risks comparable to other cohorts. This approach to anti-coagulation management deserves further direct comparison with continued warfarin therapy in randomized controlled trials.

## Data Availability

Not applicable.

## Abbreviations

ICD: Implantable cardioverter defibrillator
CIED: Cardiac implantable electronic device
AF: Atrial fibrillation
TIA: Transient ischemic attack
INR: International normalized ratio
LMWH: Low molecular weight heparin
CNS: Central nervous system
